# The road to patient experience of care measurement: lessons from the United States

**DOI:** 10.1186/2045-4015-2-35

**Published:** 2013-09-17

**Authors:** Eyal Zimlichman, Ronen Rozenblum, Michael L Millenson

**Affiliations:** 1 Sheba Medical Center, Tel Hashomer, Israel; 2 Brigham and Women’s Hospital and Harvard Medical School, Boston, MA, USA; 3 Kellogg School of Management, Evanston, IL and Health Quality Advisors LLC, Highland Park, IL, USA

**Keywords:** Patient-centered care, Patient experience, Health care policy, Israel, United States

## Abstract

Patient-centered care has become an increasing priority in the United States and plays a prominent role in recent healthcare reforms. One way the country has managed to advance patient-centered care is through establishment of a family of national patient experience surveys (the Consumer Assessment of Healthcare Providers and Systems Plans (CAHPS). CAHPS is publicly reported for several types of providers and was recently tied to hospital reimbursement. This is part of a trend over the last two decades that has shifted provider-patient relationships from a traditional paternal approach to customer service and then to clinical partnership. The health care system in Israel, however, is still struggling to overcome barriers to change in this area. While community based biannual patient experience surveys are conducted by the Myers-JDC-Brookdale Institute, there is no comprehensive national approach to measuring the patient experience across a broad range of settings. Only recently did the Israeli Ministry of Health take its first steps to include patient experience as a dimension of health care quality.

In its current position, Israel should learn from the U.S. experience with policies promoting patient-centered care, and specifically the impact on clinical services of measuring the patient experience. Looking at what has happened in the United States, we suggest three main lessons. First, there is a need for a set of national patient experience surveys that would be publicly reported and eventually tied to provider reimbursement. Secondly, the national survey tools should be customized to the unique characteristics of Israeli society and draw from recent research on patient-centeredness to include new and important domains such as patient activation and shared decision-making. Finally, newer technological approaches should be explored with the aim of increasing response rates and the timeliness and usefulness of the surveys.

## Introduction

Over the past decade, patient-centered care and patient experience have drawn increasing interest, highlighting the importance of incorporating patients’ needs and perspectives into care delivery [1,2]. Part of the impetus for implementation of patient-centered care is growing evidence that it leads to greater patient satisfaction, improved clinical outcomes, health service efficiency and a positive effect on health-related business metrics [3-8].

Consistent with this notion, patient-centered care and patient experience are considered key dimensions of healthcare quality. Widely accepted domains of patient-centered care include respect, emotional support, physical comfort, information and communication, continuity and transition, care coordination, involvement of patients and their family, and access to care [9,10]. Surveys measuring patients’ experience of health care are typically based on these domains.

Triggered in part by the 2002 IOM report, "Crossing the quality chasm: A new health system for the 21st century", patient-centered care has become an increasing priority on the national agenda in the United States and plays a prominent role in the Patient Protection and Affordable Care Act (ACA) [1,5]. Public reporting on patient experience of care surveys has further motivated healthcare organizations around the world to strive to become measurably more patient-oriented [5,11-13]. To do so, they are using a range of strategies that include redesigning and co-designing service delivery with patients, implementing patient rights charters and engaging patients as partners in improving care from the clinical level to the organizational one; i.e., “from exam room to boardroom”. Yet, many organizations continue to face barriers to transforming their culture from a provider focus to a patient-centric one, and the result is reflected in less than optimal scores on patient experience surveys [5,14,15].

Unfortunately, the healthcare system in Israel is still struggling to overcome barriers to change in this area. In spite of the emphasis on service improvement by many hospitals and health plans, the effectiveness of their efforts to improve patient experience has been mixed, a recent Israeli Ministry of Health (MOH) report showed [16]. Moreover, a recent study that examined the attitudes of clinicians from four different countries about hospital activities meant to improve patient satisfaction found a yawning gap between hospital management and frontline clinicians. Although the majority of clinicians in the United States, Denmark, the United Kingdom and Israel did not have a structured plan for improving patient satisfaction, the percentage who did in the other three countries far exceeded that in Israel. Similarly, the other three countries did much better with respect to clinicians receiving feedback from hospital management about the patient experience [14,15].

Concurrently, the Israeli MOH has taken its first steps to include patient experience as a dimension of healthcare quality, mainly by developing guidelines to improve patient experience and a national patient experience survey to assess how those guidelines are affecting actual practice. While many countries have made substantial improvements in moving towards a patient centered health care systems, such as the United Kingdom, this paper will be focusing on the learning from what has happened in the United States. From this perspective, we suggest in this paper potential directions for the Israeli healthcare system to take in improving patient-centered care, specifically through patient experience measurement.

### The emergence of patient-centered care in the United States

The United States has been a pioneer in this area, with a handful of researchers studying patient satisfaction and problems of communication as far back as the 1950s [17]. However, the current emphasis on patient engagement arose from more recent roots in the civil rights movement and feminism, on the one hand [18] – “Nothing about me without me” [19] became a patient rallying cry – and practical concerns about cost containment, on the other hand. Under President Bill Clinton’s “managed competition” proposals, the same approach which influenced Israel’s National Health Insurance Law, [20] health plans were supposed to compete for customers. But to protect against low-ball pricing that cut corners on care, third-party payers and plan members needed quality measures to balance cost ones.

U.S. government-funded researchers developed the Consumer Assessment of Health Plans Study (CAHPS), launched in 1995, with the dual aims of providing information for comparison shoppers and for internal improvement. CAHPS was the first national patient survey in which content and administration were standardized. After the Clinton administration reform effort and its emphasis on a central role of health plans collapsed, CAHPS was expanded and reborn with a new name: the Consumer Assessment of Healthcare Providers and Systems and began growing into a family of ambulatory and facilities surveys with a common methodology.

In 2006 the federal government issued the 27-item Hospital CAHPS (HCAHPS) for inpatient care. The first 22 items represent the core questions, divided into six measures/composites: nurse communication, doctor communication, responsiveness of hospital staff, pain management, communication about medicines and discharge information. The final five are demographic questions used for patient-mix adjustment. As is now true with all CAHPS surveys, it went through a review process [21] involving both government and expert stakeholder groups, such as the National Quality Forum, to produce a product that was “credible, useful and practical” [22]. In 2008, the Centers for Medicare & Medicaid Services (CMS) began posting HCAHPS results on a dedicated website in order to use public transparency and data accessibility as an extra incentive for provider self-improvement (http://www.medicare.gov/hospitalcompare). Hospitals that did not publicly report also found their reimbursement slightly reduced. The strategy seems to have worked, with a comprehensive national analysis finding “modest but meaningful gains” [23]. Aggregate CAHPS data also appear in a congressionally mandated annual National Healthcare Quality Report from the Agency for Healthcare Research and Quality.

CAHPS seeks to assess the *experience* of care by asking about behaviors the patient directly observes rather than assessing patient satisfaction. *Satisfaction* is seen as more subjective and more easily influenced by prior expectations that might be a function of age, socioeconomic status or other factors [24]. So, for instance, HCAHPS asks, “During this hospital stay, how often did doctors explain things in a way you could understand?” rather than, “How satisfied were you with your doctors?”

The National CAHPS Benchmarking Database has found persistent racial and ethnic differences, a phenomenon also present in the diverse Israeli population. However, hospital cultural competency can not only reduce those disparities “but may also contribute to general quality improvement” [25].

In addition, the line between “satisfaction” and “experience of care” is not always clear [26] due to the advent of newer terms (and new surveys designed to measure them). Among the terms are relationship-centered care, patient engagement, patient empowerment, patient activation and shared decision-making; some have a precise definition, others are still in flux. All jostle for attention under the rubric of “patient-centered care” (or, perhaps, “person-centered care” or “person- and family-centered care” or “participatory medicine”). Separately, the World Health Organization is working to make patient experience a measure of the overall “responsiveness” of the health care system.

Over time CAHPS has brought about an evolution of patient satisfaction surveys in the United States from a consumer service tool to a clinical quality monitoring tool. That has been particularly evident as CAHPS expanded to include more facilities (dialysis centers and nursing homes) and more sites of care (the Clinician & (medical) Group CAHPS and a behavioral health CAHPS). Moreover, the ACA consistently links provider and provider organization performance on a variety of publicly disclosed measures of patient-centered care directly to a portion of compensation. That’s true for hospitals under the value-based purchasing program and for hospital-clinician partnerships in new delivery forms such as the accountable care organization or the patient-centered medical home.

Meanwhile, increasing evidence is mounting about the relationship between the patient experience and medical outcomes [4,6,8]. Thus, researchers have found “unequivocal and significant relationships” between doctor-patient communication and patient outcomes such as psychological and functional status and symptom recovery [27,28]. There’s also a connection to patient medication compliance, [28] a significant problem in the Israeli health system as in other developed nations [29]. Degree of patient activation was found to be a significant predictor of cost even after adjustment for a commonly used “risk score” specifically designed to predict future costs [30], and shared decision-making can sharply lower hip and knee surgery rates and costs [31]. As a review of the evidence on patient experience measures and health outcomes recently concluded, “[B]oth theory and the available evidence suggest that such measures are robust, distinctive indicators of health care quality” [3]. Although the effect of publicly reporting patient satisfaction data is less clear, evidence has been mounting that there is an effect on consumers and provider behavior. While public reporting of comparative data on patient satisfaction has been shown to enhance and reinforce quality improvement efforts by providers [32], its effect on consumer behavior, while existent, was found to be secondary to issues such as free provider choice and costs [33].

### Shaping patient-centered care in Israel

The Israeli health care system’s path towards patient-centered care took a different course than in the United States. The four, non-profit health plans that are a major component of the Israeli health care system today are rooted in the workers’ associations that sought to provide care services to workers and their families at the beginning of the 20^th^ century [34]. The broader health care system stems from social foundations in place when the State of Israel was established in 1948. As a result, the current structure is based largely on State activities and funding sources. Although there are a few private hospitals, most acute care beds and long-term inpatient facilities are operated by the government, and the government sets the level of per capita financing that all four health plans (Clalit, Maccabi, Meuhedet and Leumit) receive. Clalit is the only health fund to run its own general hospitals, operating about one third of the general hospitals.

Israel went through its own health care reforms in the mid-1990s, instituting two landmark laws that have fundamentally impacted the patient-provider relationship: The National Health Insurance Law in 1995 and the Patient’s Rights Law in 1996. Among other things, the former ensured universal health insurance coverage for all citizens while providing free choice of health plans. The result was to increase the role of patients as consumers in a competitive market. Yet equally important was the Patient’s Rights Law, whose goals were to ensure caregiver professionalism and quality and to protect the dignity and privacy of patients [35]. The law explicitly defined the rights and obligations of patient-provider relationships, and epitomized the shift from a paternalistic model of care to a patient-centered one emphasizing patient autonomy. Specifically, the Patient’s Rights Law addressed important issues such as informed consent for treatment, provider-patient shared decision making, the right for a second opinion and more. In this, Israel was a true pioneer; U.S. law and policy, by contrast, were far more disjointed in this era.

Yet well ahead of new national laws, local initiatives have set the patient-centered care agenda. The healthcare delivery system in Israel is unique. The four non-profit health plans are partially subsidized by the government through an annual per-member capitation fee, and their competition in an open market for members has been a key driver of the patient-centered agenda. First through pilots and demonstrations, and later through a comprehensive program, all four plans now actively assess the patient experience. All have focused on measurement of primary care and specialist services in the community. At Maccabi Healthcare Services, patient experience surveys started in 1988 as a cross-sectional twice a year survey, but moved to a daily sampling of patients in 2009. Clalit Healthcare Services launched an on-going patient experience survey in 2001 for the community setting. Leumit and Meuhedet quickly followed, and all four plans now continually measure the patient experience through four different survey tools. Furthermore, all health plans have integrated patient experience into their organizational goals, setting out to become more patient-centric on all levels. Similarly, the Israel Defense Forces Medical Corps, caring for all active duty personnel, launched a community oriented (primary and secondary healthcare services) patient experience survey program in 2001. This has become the source for numerous local and centralized initiatives aimed at providing patient-centered care to soldiers [36-38].

Following the National Health Insurance Law, national surveys on experience of care were sponsored by the MOH and the health plans. They were conducted by the Myers-JDC-Brookdale Institute in Jerusalem biannually [39] via telephone surveys of a representative sample of about 1,800 adult residents. Although survey results are published, the impact on consumer behavior is unclear. Yet, the surveys most likely have had some effect on both the macro level (policy), as well as the micro level (service). One initiative attributed to the surveys were the MOH issued regulations regarding notifications on co-payments and supplemental insurance, which translated to increased publication and dissemination of relevant regulations by the health funds [40].

Measuring and comparing patient satisfaction with hospital care in Israel is more sporadic, and results of surveys are rarely available publically [16,41]. While Clalit did initiate an on-going patient experience survey for its hospitals in 2001, most other general, as well as rehabilitation and post-acute care facilities, have not done so. A 2011 survey comparing all government-owned hospitals on patient experience of care was criticized in the media as not addressing the right questions and not being adequately transparent [42]. With that in mind, the MOH has recently committed to institute on-going, publicly reported, national surveys for patient experience aimed initially at acute-care hospitals, starting in 2013/2014, and later other inpatient settings and community care.

### What can Israel learn from the U.S. experience?

As policy in Israel is being shaped, what can Israel learn from the United States in regards to measuring the patient experience? First of all, we believe there is a clear need for a comprehensive set of national patient experience surveys (including all settings of care) in Israel as an important first step in moving down the road toward a more comprehensive patient-centered care agenda. Prioritizing this effort we would suggest starting with an inpatient patient experience survey in general hospitals, since it seems that the biggest gap (and maybe the potential for improvement) is currently for assessing patient experience within hospitals. This should be followed with other settings that would include out-patient care, psychiatric hospitals, rehabilitation and geriatric and long-term care facilities. While today there is a national biannual community patient experience survey (supervised through Myers-JDC-Brookdale), this needs to be either more frequent or through on-going sampling, similar to the HCAHPS survey in the United States. On-going sampling enables providers to better respond to changes in patient experience trends, while minimizing effects of seasonality or one-time events. Furthermore, policy makers in Israel should consider having survey results be transparent to the general public as they are in the United States. While this seems to have provoked provider interest in real improvement in the U.S., cultural differences might prove to be significant enough for transparency to deliver different outcomes in Israel. Publicly reporting survey results could drive higher quality health care services mostly by allowing providers to identify areas for improvement through benchmarking and also by leveraging public pressure. Public reporting could also increase competition by promoting choice for patients who would now have better data on which to base their decisions.

Secondly, we believe that the unique characteristics of Israeli society require a customized set of tools rather than off-the-shelf use of tools developed for other nations. Certainly, an Israeli tool should still be based on current validated questions that would be adjusted through Israeli focus groups and pilot surveys. Moreover, Israel has the opportunity to take advantage of research on patient-centeredness conducted since the U.S. and other surveys were developed. An Israeli patient experience survey could also move away from traditional experience measures such as satisfaction with meals or beds and encompass new and important domains such as patient activation and shared decision-making. Although current Israeli policymakers are still not, in our view, adequately encouraging patient empowerment and patient-centered care, we expect that the same strong cultural, clinical and economic trends that have brought this to center stage in the United States will bring change to Israel in just a few years. Early evidence shows that Israeli patients are interested in being more involved in their care decisions [43].

Finally, new and innovative solutions need to be considered for measuring the patient experience. U.S. hospitals conduct the HCAHPS, mostly through vendors, and report results to CMS. However, the surveys are predominantly conducted through regular mail, post-hospitalization, and response rates that traditionally have been in the 30-40% range, raising doubts about potential response and selection bias in the results. As Israel launches its own hospital patient experience survey, newer technological approaches should be explored, including telephone interviews, Internet surveys, interactive voice response (IVR) and smartphone applications, all with the aim of increasing response rates, timeliness and usefulness, while taking any potential bias into consideration. The very high penetration of Internet and of health plan patient portals in the Israeli population introduces another opportunity to collect patient-reported data more efficiently and in a more customized manner than in the United States.

Still, when attempting to draw lessons from the U.S. experience in encouraging patient-centered care, one must take into account several fundamental societal differences. As mentioned previously, the Israeli system was built upon a foundation of social solidarity among citizens that is symbolized by the government’s responsibility to provide universal health insurance coverage for all. The U.S. system was built on a foundation of doctors and hospitals as independent businesses, with private health insurance linked to employment for those not involved in government programs for the elderly or the poor. That system, whatever its drawbacks, includes an element of competition for consumers that is now evolving from a focus on service items (parking and hospital amenities) to a focus on more substantive elements, such as genuine patient-centered care. Inherent in those differences is the greater choice for patients in selection of service providers the U.S. health care system is providing while the Israeli system has much less choice of providers. This has contributed to creating a system in Israel that traditionally has been less patient-centered. Yet legislation such as the National Health Insurance Law and the Patient’s Rights Law together with the four health plans system have empowered the patient and contributed to putting the Israeli system on the threshold of being much more patient–oriented than in the past. Furthermore, the gradual but consistent proliferation of the private health insurance market in Israel is contributing to increased choice of health care providers by patients and encourages both public and private providers to focus more on patient experience.

An important second difference lies in the decisions made about resource allocation. Health care leadership in Israel, especially at hospitals, is limited in its ability to make changes in the bed complement, upgrade facilities, add or fire personnel, add or drop services or equipment, etc. In a tacit acknowledgement of that reality, many policymakers and health system leaders have opposed assessing the patient experience of care because of expectations that the results will highlight the system’s shortcomings while leaving them few ways in which to respond. Nonetheless, because patient-centered care goes beyond simple patient experience surveys, it has slowly edged its way onto the national agenda, even if later than most other developed countries, including the United States.

## Conclusions

Creating a patient-centered health care system demands more than just measurement, yet measurement must be part of a comprehensive national strategy. In Israel, the foundation has been laid for this change through the fundamental legislation that shaped the system. Meanwhile, the health plans have already started to move in this direction.

But more action is now needed from the Israeli Ministry of Health. Drawing on the U.S. experience, this should include launching a comprehensive set of national patient experience surveys across all settings, albeit one customized to Israeli culture and needs; public reporting of results by institution; and payment incentives, such as value-based purchasing, that encourage performance improvement and promote the patient-centered care that each and every Israeli citizen deserves.

## Competing interests

EZ and RR are both members of the Israeli Ministry of Health-appointed steering committee for evaluation of patient experience in hospitals and the community. They both report no other potential competing interests.

MLM reports no potential competing interests.

## Authors’ contributions

All authors participated in the discussions leading to the written conclusions, have participated in drafting the manuscript and have read and approved the final manuscript.

## Authors’ information

EZ is an internal medicine physician, the Deputy Director and Chief Quality Officer at Sheba Medical Center, Tel Hashomer, Israel and is an affiliated researcher with Brigham and Women’s Hospital, Partners Healthcare System and Harvard Medical School, all in Boston, MA, USA. Until recently EZ has been a lead researcher at Partners Healthcare’s department of Clinical Affairs.

RR is the Director of the Unit for Innovative Healthcare Practice & Technology, The Center for Patient Safety Research and Practice, Brigham and Women’s Hospital and Harvard Medical School, Boston USA. RR is also a leading researcher in the field of patient-centered care and patient experience at Harvard Medical School where he holds a faculty position as Instructor in Medicine.

MLM is the Mervin Shalowitz, MD Visiting Scholar at Northwestern University’s Kellogg School of Management, Evanston, IL USA and president of Health Quality Advisors LLC, Highland Park, IL USA.
